# Therapeutic hypothermia in traumatic brain injury

**DOI:** 10.1186/cc11269

**Published:** 2012-06-07

**Authors:** Jonathan KJ Rhodes

**Affiliations:** 1 University of Edinburgh, Department of Anaesthesia, Critical Care and Pain Medicine, Intensive Care Unit, Western General Hospital, Edinburgh, UK

## 

Hypothermia has profound effects on the brain function but importantly is potentially protective against both focal and global injuries. Aspects of the biochemical response to acute ischaemia and trauma, which are associated with poor outcome, can be inhibited by cooling. Unlike many pharmacological treatments that tend to antagonise a single neurochemical process, hypothermia offers a simple method of inhibiting multiple pathological processes simultaneously. It therefore has the potential, if applied correctly, to improve outcomes after acute brain injuries, where drug trials have so far failed.

The systemic cooling of patients after acute brain injury is an established treatment modality in many neuro-ICUs. It is a strategy for protecting the injured brain that makes intuitive sense and can reduce both intracranial pressure and the potential for ischaemic secondary insults. Basic science evidence also suggests that cooling can attenuate many secondary biochemical cascades that are activated after acute injury.

However, despite these multiple lines of supportive evidence there is as yet no confirmation from a high-quality randomised controlled trial that prophylactic hypothermia improves outcome or reduces mortality.

This talk will look at the potentially beneficial effects of hypothermia on the biochemistry of acute brain injury, consider the reasons for the failure to demonstrate clinical efficacy and review the supportive data from meta-analysis, suggesting how hypothermia might be best delivered. Finally I will discuss EuroTherm3235, a European Society of Intensive Care Medicine funded multicentre randomised controlled trial investigating prophylactic hypothermia in traumatic brain injury, which draws on the lessons from the available literature.
